# Metformin inhibits IL-1β secretion via impairment of NLRP3 inflammasome in keratinocytes: implications for preventing the development of psoriasis

**DOI:** 10.1038/s41420-020-0245-8

**Published:** 2020-03-04

**Authors:** Gaku Tsuji, Akiko Hashimoto-Hachiya, Vu Hai Yen, Masaki Takemura, Ayako Yumine, Kazuhisa Furue, Masutaka Furue, Takeshi Nakahara

**Affiliations:** 1 Research and Clinical Center for Yusho and Dioxin, Kyushu University, Maidashi 3-1-1, Higashiku, Fukuoka, 812-8582 Japan; 2 Department of Dermatology, Graduate School of Medical Sciences, Kyushu University, Maidashi 3-1-1, Higashiku, Fukuoka, 812-8582 Japan; 3 Division of Skin Surface Sensing, Graduate School of Medical Sciences, Kyushu University, Maidashi 3-1-1, Higashiku, Fukuoka, 812-8582 Japan

**Keywords:** Inflammasome, Skin diseases

## Abstract

Psoriasis is a systemic inflammatory disease significantly associated with comorbidities including type 2 diabetes mellitus (T2DM). Metformin is utilized as a first-line agent for treating T2DM. Although metformin reportedly inhibits mature IL-1β secretion via NLRP3 inflammasome in macrophages of T2DM patients, it remains unclear whether it affects skin inflammation in psoriasis. To test this, we analysed normal human epidermal keratinocytes (NHEKs), a major skin component, stimulated with the key mediators of psoriasis development, TNF-α and IL-17A. This stimulation induced the upregulation of pro-IL-1β mRNA and protein levels, and subsequently mature IL-1β secretion, which was inhibited by metformin treatment. To further reveal the mechanism involved, we examined how metformin treatment affected NLRP3 inflammasome activated by TNF-α and IL-17A stimulation. We found that this treatment downregulated caspase-1 expression, a key mediator of NLRP3 inflammasome. Furthermore, inhibitors of AMPK and SIRT1 abrogated the downregulation of caspase-1 induced by metformin treatment, indicating that AMPK and SIRT1 are essential for the inhibitory effect on NLRP3 inflammasome in NHEKs. As IL-1β stimulation induced upregulation of IL-36γ, CXCL1, CXCL2, CCL20, S100A7, S100A8 and S100A9 mRNA and protein levels in NHEKs, we examined whether metformin treatment affects such gene expression. Metformin treatment inhibited upregulation of IL-36γ, CXCL1, CXCL2, CCL20, S100A7, S100A8 and S100A9 mRNA and protein levels induced by TNF-α and IL-17A stimulation. Finally, we examined whether metformin administration affected psoriasis development in an imiquimod-induced mouse psoriasis model. Oral metformin treatment significantly decreased ear thickness, epidermal hyperplasia and inflammatory cell infiltration. A cytokine profile in the epidermis under metformin treatment showed that IL-1β, Cxcl1, Cxcl2, S100a7, S100a8 and S100A9 mRNA levels were downregulated compared with control levels. These results indicate that metformin administration prevented psoriasis development in vivo. Collectively, our findings suggest that metformin-mediated anti-psoriatic effects on the skin have the potential for treating psoriasis in T2DM patients.

## Introduction

Psoriasis is a common chronic inflammatory disease of the skin affecting 0.6–3% of the global population^1–3^. The skin lesions in psoriasis manifest as scaly multiple erythema on the face, scalp, trunk and upper and lower extremities, which places a significant physical and psychological burden on affected patients, resulting in a decreased quality of life^4^. Psoriasis frequently coexists with other systemic diseases such as type 2 diabetes mellitus (T2DM)^5–8^, arterial hypertension^9,10^, obesity^11–13^ and cardiovascular disease^14–16^. Several retrospective epidemiological studies have shown the association of T2DM with psoriasis and suggested that there is a severity-dependent relationship^7,8^. Therefore, the management of T2DM is very important in the treatment of psoriasis.

Metformin is utilized as a first-line antidiabetic agent for the treatment of T2DM, as it has been shown to achieve additional beneficial effects such as weight reduction, decreased hyperinsulinemia, improved lipid profiles and decreased cardiovascular risk in T2DM patients^17–20^. One clinical study has shown that the long-term use of metformin was associated with a reduced risk of psoriasis^21^. Moreover, metformin treatment has been shown to improve the disease severity in psoriasis patients^22,23^. These lines of clinical evidence suggest that metformin treatment has dual beneficial effects on psoriasis and T2DM. It has also been shown that NLRP3 inflammasome, a master regulatory system of mature interleukin (IL)-1β secretion^24^, is highly activated in monocyte-derived macrophages from patients with T2DM, and that metformin treatment reduces the secretion of mature IL-1β^25^. It is well-established that the precursor form pro-IL-1β is not biologically active and requires posttranslational processing for activation. Specifically, upon stimulation, inactive pro-IL-1β is cleaved into mature IL-1β by caspase-1, which is induced by NLPR3 inflammasome activation in the skin^24^.

In the epidermis of skin lesions of psoriasis patients, IL-1β has been shown to be increased^26,27^ and effective treatment of psoriasis led to a significant decrease in epidermal IL-1β expression^28^. In addition, IL-1α expression is decreased, whereas IL-1β expression is increased in the lesional skin of psoriasis patients^29,30^. Furthermore, caspase-1 expression is reportedly upregulated in psoriatic skin^27^. These lines of evidence led us to hypothesize that metformin treatment may modulate mature IL-1β secretion via NLRP3 inflammasome in the skin, resulting in the prevention of psoriasis development.

The recent therapeutic success of anti-tumour necrosis factor (TNF)-α and anti-IL-17 antibodies has emphasized the critical roles of TNF-α and IL-17A in psoriasis development^31,32^. Therefore, we tested whether metformin treatment modifies the secretion of mature IL-1β mediated by NLRP3 inflammasome in TNF-α- and IL-17A-stimulated normal human epidermal keratinocytes (NHEKs), a major component of the skin. We found that metformin treatment inhibited the upregulation of pro-IL-1β mRNA and protein levels, and mature IL-1β secretion. We also found that metformin treatment downregulated caspase-1 expression, resulting in reduced mature IL-1β secretion in NHEKs, via a mechanism that was dependent on SIRT1, a nicotinamide adenine dinucleotide-dependent deacetylase and a target molecule activated by metformin^33^.

Furthermore, IL-1β stimulation of NHEKs was found to induce upregulation of the mRNA and protein levels of pro-inflammatory cytokines, chemokines and antibiotic peptides including IL-36γ, CXCL1, CXCL2, CCL20, S100A7, S100A8 and S100A9, which are closely related to the pathogenesis of psoriasis.

IL-36γ, an IL-1F cytokine formerly known as IL-1 F9, is reportedly highly expressed in the epidermis of psoriasis skin lesions. Peripheral blood serum levels of IL-36γ are also closely associated with disease activity and decline under TNF-α treatment^34^, suggesting that IL-36γ is a valuable biomarker in psoriasis patients for evaluating disease activity during the clinical course.

CXCL1^35^, CXCL2^36^ and CCL20^37^, which are expressed in psoriatic tissue, chemoattract neutrophils and IL-17A-producing T cells from the blood into inflamed cutaneous tissue. Therefore, CXCL1, CXCL2 and CCL20 contribute to a positive chemotactic feedback loop recruiting inflammatory cells into psoriatic lesions.

S100A7, S100A8 and S100A9, calcium-binding proteins, are located in the psoriasis susceptibility locus 4 and are highly expressed at the epidermis of psoriasis skin lesions^38^. It has been reported that a mouse model with the overexpression of S100A7 displayed psoriasis-like symptoms^39^, indicating that S100A7 proteins are potential mediators of psoriasis. The expression of S100A8–S100A9 reportedly lead to uncontrolled immune cell activation, angiogenesis, hyperproliferation of keratinocytes and, finally, the chronic inflammation that typifies psoriasis. In addition, loss of S100A8–S100A9 led to prevention of the psoriasis-like phenotype^38^, which indicates that S100A8–S100A9 are potential therapeutic targets for psoriatic patients.

Therefore, we examined whether metformin treatment affected the expression of these genes in TNF-α- and IL-17A-stimulated NHEKs, and found that it inhibited the upregulation of IL-1β, IL-36γ, CXCL1, CXCL2, CCL20, S100A7, S100A8 and S100A9 mRNA and protein levels induced by TNF-α and IL-17A stimulation. Finally, we examined whether metformin treatment affected the development of psoriasis in an imiquimod (IMQ)-induced mouse psoriasis model, a well-established murine psoriasiform dermatitis model^40,41^. Oral metformin treatment significantly improved the clinical features, with reduced expression of *IL-1β*, *Cxcl1*, *Cxcl2*, *S100a7*, *S100a8* and *S100a9* in the epidermis. Collectively, our findings suggest that metformin-mediated anti-psoriatic effects on the skin could be utilized for treating psoriasis patients with T2DM.

## Materials and methods

### Reagents and antibodies

Human recombinant TNF-α, IL-17A and IL-1β were purchased from PeproTech (Rocky Hill, NJ, USA). Dimethyl sulfoxide (DMSO) was purchased from Nacalai Tesque (Kyoto, Japan). Sirtinol (Enzo Life Science, Farmingdale, NY, USA) and dorsomorphin (Abcam, Cambridge, UK) were dissolved in DMSO and stored at −30 °C until used in the experiments. Metformin (Metformin hydrochloride, Tokyo Chemical Industry, Tokyo, Japan) was dissolved in medium and filtrated by 0.22 µm polyvinylidene difluoride (PVDF) membrane (Merck Millipore, Burlington, MA, USA). Anti-human pro-IL-1β monoclonal rabbit antibody, anti-human NLRP3 monoclonal rabbit antibody, anti-human caspase-1 monoclonal rabbit antibody, anti-human β-actin monoclonal mouse antibody, anti-phosphorylated AMPKα rabbit monoclonal antibody (Thr172), anti-AMPKα rabbit monoclonal antibody, anti-human S100A9 monoclonal rabbit antibody (Cell Signaling Technology, Danvers, MA, USA), anti-human IL-36γ monoclonal mouse antibody, anti-human S100A7 monoclonal mouse antibody and anti-human S100A8 monoclonal rabbit antibody (Abcam) were used for western blotting.

### Cell culture

NHEKs obtained from Lonza (Basel, Switzerland) were grown in culture dishes at 37 °C in 5% CO_2_. They were cultured in serum-free keratinocyte growth medium (KGM-gold, Lonza) supplemented with bovine pituitary extract, recombinant epidermal growth factor, insulin, hydrocortisone, transferrin and epinephrine. Culture medium was replaced every 2–3 days. Cells approaching confluence (70–90%) were disaggregated with 0.25 mg/mL trypsin/0.01% ethylenediaminetetraacetic acid and subcultured. Second- to fourth-passage NHEKs were used in all experiments. NHEKs were seeded, allowed to attach for 24 h and then utilized for further experiments.

### Cell viability assay

To evaluate cell viability, we utilized the WST-1 cell proliferation assay system (Takara Bio, Shiga, Japan), in accordance with the manufacturer’s protocol. Optical density was measured using a DTX 800 Multimode Detector (Beckman Coulter, Brea, CA, USA). The cell viability results are presented in Supplementary Fig. 1.

### Quantitative real-time PCR

Total RNA was extracted using the RNeasy Mini kit (Qiagen, Hilden, Germany). Reverse transcription was performed using PrimeScript RT-reagent kit (Takara Bio). Quantitative real-time PCR (qRT-PCR) was performed on the CFX Connect Real-time System (Bio-Rad, Hercules, CA, USA) using TB Green Premix Ex Taq (Takara Bio). Amplification was started at 95 °C for 30 s as the first step, followed by 40 cycles of qRT-PCR at 95 °C for 5 s and 60 °C for 20 s. mRNA expression was measured in triplicate and was normalized using the β-actin expression levels. The primer sequences are shown in Supplementary Fig. 2.

### Western blotting analysis

NHEKs were incubated for 5 min in lysis buffer (Complete Lysis M; Roche Diagnostics, Rotkreuz, Switzerland). The lysate protein concentration was measured with a BCA protein assay kit (Thermo Scientific, Waltham, MA, USA). Equal amounts of protein were dissolved in NuPage LDS sample buffer (Thermo Fisher Scientific) and 10% NuPage sample reducing agent (Invitrogen). Lysates were heated at 70 °C for 10 min and loaded and run on Bis-Tris Gel (Thermo Fisher Scientific) at 200 V for 20 min. The proteins were transferred to PVDF membrane (Merck Millipore, Burlington, MA, USA) and blocked in Western breeze blocker/diluent (Thermo Fisher Scientific). Membranes were probed with primary antibody or anti-β-actin antibody overnight at 4 °C. Anti-mouse and anti-rabbit horseradish peroxidase-conjugated IgG antibodies (Cell Signaling Technology) were used as secondary antibodies. Visualization of protein bands was accomplished with Super Signal West Pico Plus Chemiluminescent Substrate (Thermo Fisher Scientific) using the ChemiDoc Touch Imaging System (Bio-Rad).

### Enzyme-linked immunosorbent assay

IL-1β, CXCL1, CXCL2 and CCL20 ELISA kits (R&D Systems, Minneapolis, MN, USA) were used in accordance with the manufacturer’s protocols. Optical density was measured using a DTX 800 Multimode Detector (Beckman Coulter).

### Mice

Four to 6 weeks old female C57BL/6 mice were obtained from the Kyushu University Animal Production Program, housed in a clean facility, and bred and used in accordance with institutional guidelines. The Kyushu University Animal Research Committee approved all of our experiments.

### IMQ‐induced psoriasis mouse model

The skin of the left ear of each mouse was treated daily for 3 or 5 days with 62.5 mg of 5% IMQ cream (Beselna cream; Mochida Pharmaceutical, Tokyo, Japan). Ear thickness was measured using a dial thickness gauge.

### Metformin administration

Metformin was dissolved in sterile distilled water and administered (100 mg/kg, once daily or 200 mg/kg once daily) to mice by gavage for 3 or 5 days.

### Histology and immunohistochemistry

The ear of each killed mouse was excised and fixed in 10% buffered formalin phosphate. Then, samples were paraffin‐embedded, sectioned and either stained with haematoxylin and eosin or prepared for IL-17A immunohistochemistry. Anti-murine IL-17A rabbit polyclonal antibody was purchased from Thermo Fisher Scientific.

For immunohistochemistry, sections were deparaffinized and hydrated by washing them in xylene followed by a graded alcohol series. Then, sections were incubated in 10 mM citric acid (pH 6) at 100 °C for 40 min. Next, they were blocked for 60 min at room temperature, followed by incubation with anti-murine IL-17A rabbit polyclonal antibody overnight at 4 °C. Samples were washed and incubated for 60 min with secondary anti-rabbit antibody (Abcam) and developed using VECTOR^®^ Red Alkaline Phosphatase (Red AP) Substrate Kit (Vector Laboratories, Burlingame, CA, USA). All stained sections were counterstained with haematoxylin. Skin sections from six mice per group were stained and photomicrographs were taken of representative fields at a magnification of ×400. Quantification of cells stained for IL-17A in ×400 magnified fields of the dermis was performed in a blinded manner (three fields were analysed per skin section).

### Epidermal RNA extraction

Mouse ears were separated into dorsal and ventral halves and incubated for 20 min at 37 °C on 3.8% ammonium thiocyanate (Sigma-Aldrich, St. Louis, MI, USA) to collect the epidermal sheet from the ears. This sheet immersed in TRIZOL (Thermo Fischer Scientific) was homogenized by FastPrep homogenizer (MP Biomedicals, LLC, Irvine, CA, USA). Then, mRNA was purified using RNeasy Mini Kit (Qiagen, Santa Clarita, CA, USA).

### Statistical analysis

Unpaired Student’s *t*-test (when two groups were analysed) and one-way analysis of variance (for three or more groups) were used to analyse the results, with a *p*-value < 0.05 being considered to indicate a statistically significant difference.

## Results

### Metformin treatment inhibited TNF-α- and IL-17A-induced IL-1β secretion in NHEKs

**Fig. 1:**
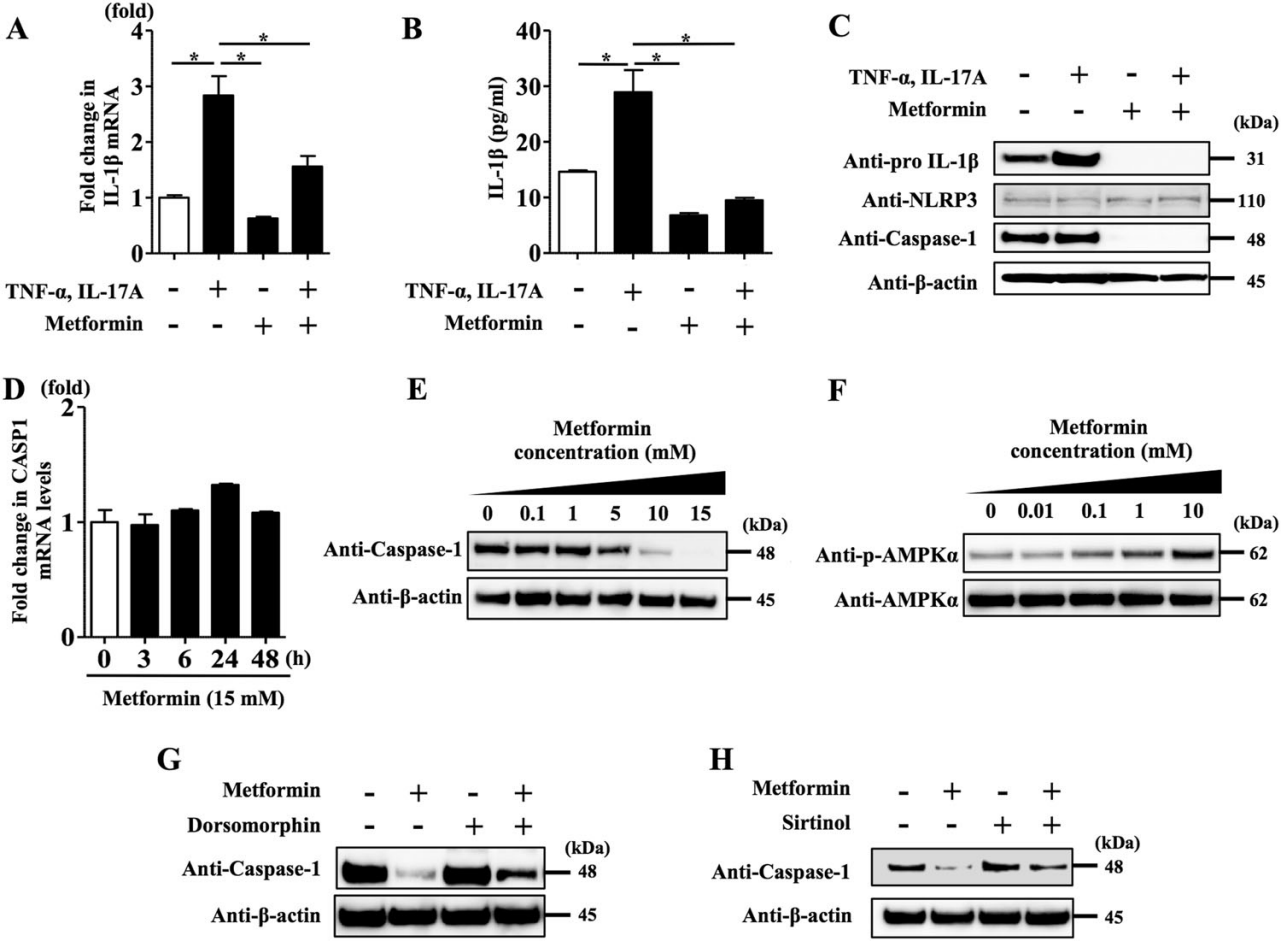
Metformin treatment inhibited TNF-α- and IL-17A-induced IL-1β secretion via impairment of NLRP3 inflammasome in NHEKs. NHEKs were stimulated with TNF-α (10 ng/ml) and IL-17A (10 ng/ml) for 24 h in the presence or absence of metformin (15 mM). **a** Pro-IL-1β mRNA level was analysed by qRT-PCR. **b** IL-1β secretion (mature IL-1β) in culture supernatant was measured using ELISA. **c** Total cell lysates were prepared and subjected to western blotting analysis with an anti-pro-IL-1β antibody, an anti-NLRP3 antibody and an anti-caspase-1 antibody. The data are representative of experiments repeated three times with similar results. **d** NHEKs were treated with metformin (15 mM) for 3, 6, 24 and 48 h. Caspase-1 mRNA level was analysed by qRT-PCR. **e** NHEKs were treated with metformin (0.1, 1, 10 and 15 mM) for 24 h. Total cell lysates were prepared and subjected to western blotting analysis with an anti-caspase-1 antibody. **f** NHEKs were treated with metformin (0.01, 0.1, 1 and 10 mM) for 3 h. Total cell lysates were prepared and subjected to western blotting analysis with an anti-phosphorylated AMPKα and an anti-AMPKα antibody. NHEKs were treated with metformin (15 mM) for 24 h in the presence or absence of dorsomorphin (1 μM) (**g**) or sirtinol (5 μM) (**h**). Total cell lysates were prepared and subjected to western blotting analysis with an anti-caspase-1 antibody. **a**, **b**, **d** Data are expressed as mean ± SEM; *n* = 3 for each group. *P < 0.05. **c**, **e**–**h** The data are representative of experiments repeated three times with similar results.

We analysed NHEKs stimulated with TNF-α (10 ng/ml) and IL-17A (10 ng/ml) for 24 h in the presence or absence of metformin (15 mM). qRT-PCR analysis revealed that metformin inhibited upregulation of the pro-IL-1β mRNA level induced by TNF-α and IL-17A stimulation (Fig. 1a). Enzyme-linked immunosorbent assay (ELISA) analysis of the culture medium also showed that metformin treatment inhibited IL-1β secretion (mature IL-1β) induced by TNF-α and IL-17A stimulation (Fig. 1b).

We further examined whether metformin treatment affected NLRP3 inflammasome activation, a master regulatory system for IL-1β secretion^24^. To test this, we analysed NHEKs stimulated with TNF-α (10 ng/ml) and IL-17A (10 ng/ml) for 24 h in the presence or absence of metformin (15 mM) by western blotting using anti-pro-IL-1β antibody, anti-NLRP3 antibody and anti-caspase-1 antibody. Metformin treatment reduced the level of pro-IL-1β protein induced by TNF-α and IL-17A stimulation (Fig. 1c). Although the NLRP3 protein level was not altered by either TNF-α and IL-17A stimulation or metformin treatment, we found that metformin treatment downregulated the caspase-1 protein level (Fig. 1c). To further reveal the mechanism involved in this, we analysed caspase-1 mRNA expression in NHEKs treated with metformin (15 mM) for 3, 6, 24 and 48 h using qRT-PCR; however, metformin treatment did not change caspase-1 mRNA expression (Fig. 1d). Moreover, western blotting analysis on the caspase-1 protein level in NHEKs treated with metformin (0.1–15 mM) for 24 h revealed that metformin treatment downregulated caspase-1 protein level in a dose-dependent manner (Fig. 1e).

These findings indicate that metformin treatment has multiple inhibitory effects on TNF-α- and IL-17A-induced pro-IL-1β synthesis (Fig. 1a, c) and IL-1β secretion (mature IL-1β) (Fig. 1b) via the impairment of NLRP3 inflammasome activation (Fig. 1c, d) in NHEKs.

A recent study has shown that the AMPK-SIRT1 axis can modulate NLRP3 inflammasome activation^42^. Therefore, we examined whether metformin treatment decreased caspase-1 expression using inhibitors of AMPK and SIRT1 in NHEKs. Metformin has been identified as a potent AMPK activator^43^. We examined whether treatment with it induced AMPK phosphorylation in NHEKs, with the results showing that it induced such phosphorylation in a dose-dependent manner (Fig. 1f). To examine whether AMPK phosphorylation is involved in the mechanism by which metformin treatment downregulates caspase-1 expression, we analysed NHEKs treated with metformin (15 mM) for 24 h in the presence or absence of dorsomorphin, an inhibitor of AMPK activity^44^. Dorsomorphin (1 μM) prevented downregulation of the caspase-1 protein level induced by metformin treatment (Fig. 1g).

Next, we focused on the involvement of SIRT1, as metformin has also been shown to potentiate SIRT1 activity^33^. We examined whether sirtinol, a SIRT1 inhibitor^45^, reversed the downregulation of caspase-1 induced by metformin treatment. Western blotting analysis of NHEKs treated with metformin (15 mM) for 24 h in the presence or absence of sirtinol (5 μM) showed that sirtinol incubation blocked the downregulation of caspase-1 induced by metformin treatment (Fig. 1h). Treatment with metformin (15 mM), dorsomorphin (1 μM) and sirtinol (5 μM) for 24 h did not affect the viability of NHEKs (Supplementary Fig. 1). These findings suggest that metformin treatment downregulated caspase-1 expression via AMPK and SIRT1 activation in NHEKs.

### IL-1β stimulation induced upregulation of IL-36γ, CXCL1, CXCL2, CCL20, S100A7, S100A8 and S100A9 expression in NHEKs

**Fig. 2:**
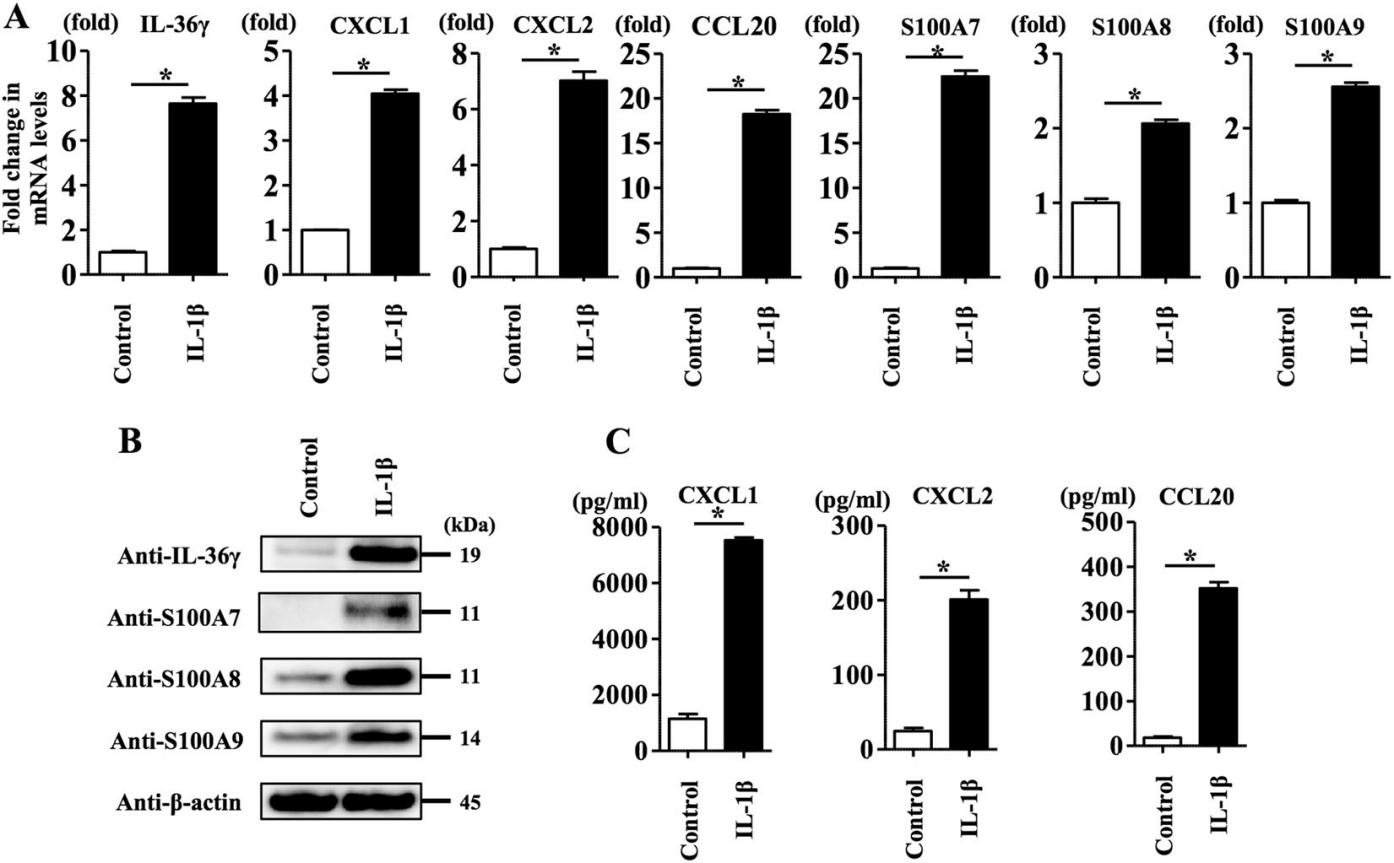
IL-1β stimulation induced upregulation of IL-36γ, CXCL1, CXCL2, CCL20, S100A7, S100A8 and S100A9 expression in NHEKs. NHEKs were stimulated with IL-1β (10 ng/ml) for 24 h. **a** IL-36γ, CXCL1, CXCL2, CCL20, S100A7, S100A8 and S100A9 mRNA levels were analysed by qRT-PCR. Data are expressed as mean ± SEM; *n* = 3 for each group. *P < 0.05. **b** Total cell lysates were prepared and subjected to western blotting analysis with anti-IL-36γ, anti-S100A7, anti-S100A8 and anti-S100A9 antibodies. The data are representative of experiments repeated three times with similar results. CXCL1, CXCL2 and CCL20 production in culture supernatant was measured using ELISA. **a**, **c** Data are expressed as mean ± SEM; *n* = 3 for each group. *P < 0.05.

To further reveal how the inhibitory effect of metformin treatment on IL-1β secretion (mature IL-1β) modulates the development of psoriasis, we focused on IL-1β-inducible pro-inflammatory cytokines, chemokines and antibiotic peptides including IL-36γ, CXCL1, CXCL2, CCL20, S100A7, S100A8 and S100A9, which are closely related to the development of psoriasis^34–39^. To test this in NHEKs, we analysed IL-1β (10 ng/ml)-stimulated NHEKs for 24 h. qRT-PCR analysis and western blotting analysis showed IL-1β stimulation induced the upregulation of IL-36γ, CXCL1, CXCL2, CCL20, S100A7, S100A8 and S100A9 mRNA levels (Fig. 2a) and protein levels of IL-36γ, S100A7, S100A8 and S100A9 (Fig. 2b). ELISA analysis of the culture media also showed that IL-1β (10 ng/ml) stimulation induced CXCL1, CXCL2 and CCL20 secretion (Fig. 2c).

### Metformin treatment inhibited TNF-α- and IL-17A-induced upregulation of IL-36γ, CXCL1, CXCL2, CCL20, S100A7, S100A8 and S100A9 expression in NHEKs

**Fig. 3:**
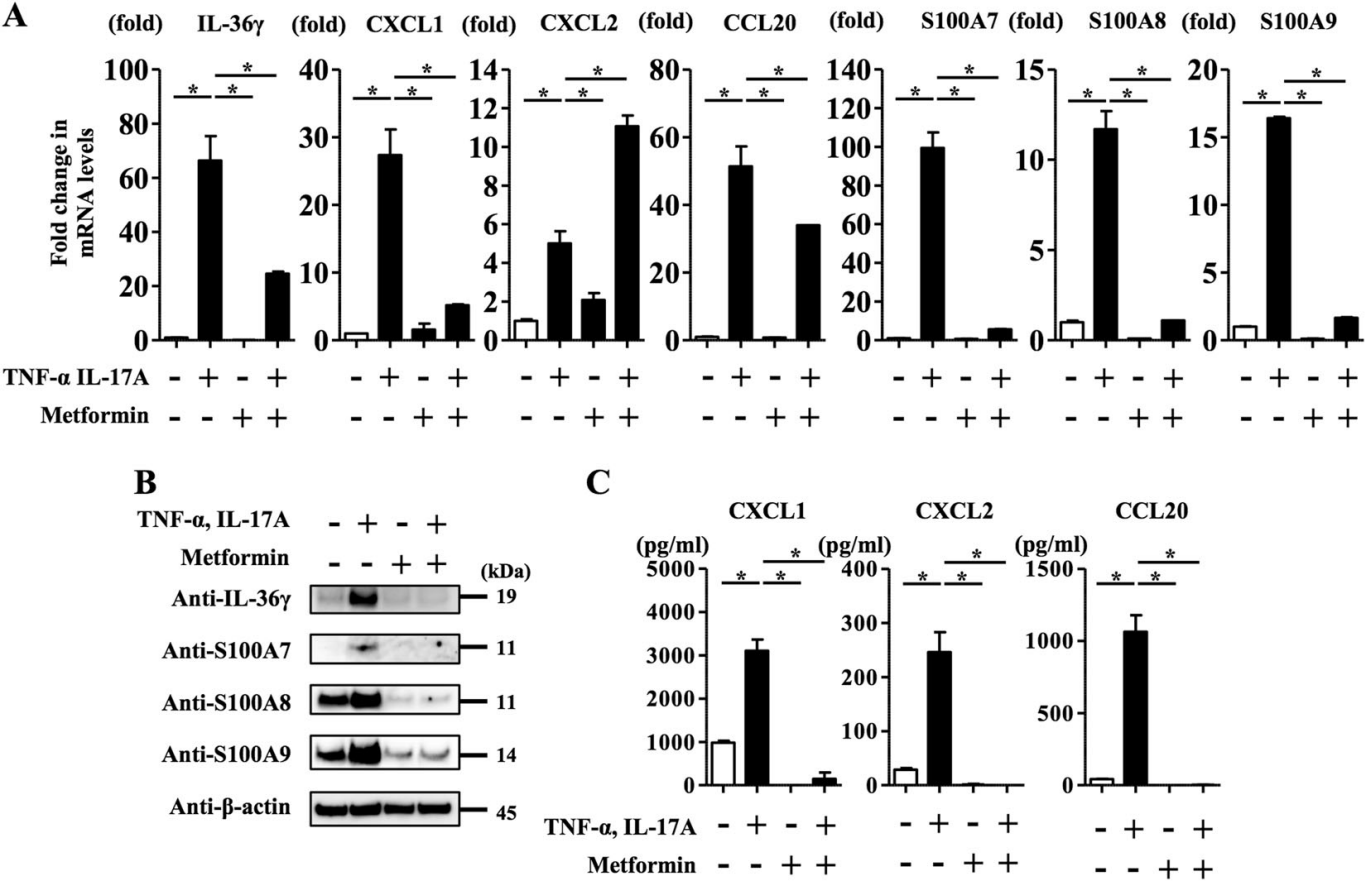
Metformin treatment inhibited TNF-α- and IL-17A-induced upregulation of IL-36γ, CXCL1, CXCL2, CCL20, S100A7, S100A8 and S100A9 expression in NHEKs. NHEKs were stimulated with TNF-α (10 ng/ml) and IL-17A (10 ng/ml) for 24 h in the presence or absence of metformin (15 mM). **a** IL-36γ, CXCL1, CXCL2, CCL20, S100A7, S100A8 and S100A9 mRNA levels were analysed by qRT-PCR. **b** Total cell lysates were prepared and subjected to western blotting analysis with anti-IL-36γ, anti-S100A7, anti-S100A8 and anti-S100A9 antibodies. The data are representative of experiments repeated three times with similar results. **c** CXCL1, CXCL2 and CCL20 production in culture supernatant was measured using ELISA. **a**, **c** Data are expressed as mean ± SEM; *n* = 3 for each group. *P < 0.05.

Next, we examined whether metformin treatment affected the expression of IL-1β-inducible pro-inflammatory cytokines, chemokines and antibiotic peptides including IL-36γ, CXCL1, CXCL2, CCL20, S100A7, S100A8 and S100A9. We analysed NHEKs stimulated with TNF-α (10 ng/ml) and IL-17A (10 ng/ml) for 24 h in the presence or absence of metformin (15 mM). As shown in Fig. 1b, metformin inhibited the IL-1β secretion (mature IL-1β) induced by TNF-α (10 ng/ml) and IL-17A (10 ng/ml). qRT-PCR analysis revealed that metformin inhibited upregulation of IL-36γ, CXCL1, CCL20, S100A7, S100A8 and S100A9 mRNA levels induced by TNF-α and IL-17A stimulation (Fig. 3a). Western blotting analysis of NHEKs stimulated with TNF-α (10 ng/ml) and IL-17A (10 ng/ml) for 24 h in the presence or absence of metformin (15 mM) confirmed that metformin treatment reduced IL-36γ, S100A7, S100A8 and S100A9 protein levels induced by TNF-α and IL-17A stimulation (Fig. 3b). ELISA analysis of the culture media also showed that metformin treatment inhibited CXCL1, CXCL2 and CCL20 production induced by TNF-α and IL-17A stimulation (Fig. 3c). Considering the finding that CXCL2 and CCL20 protein levels in the culture media were reduced by metformin treatment in TNF-α- and IL-17A-stimulated NHEKs (Fig. 3c), the reason why CXCL2 mRNA level was preferentially increased by metformin treatment in TNF-α and IL-17A-stimulated NHEKs (Fig. 3a) is likely to be associated with the timing of mRNA collection from NHEKs.

### Oral metformin administration prevented the development of IMQ-induced psoriasiform eruptions

**Fig. 4:**
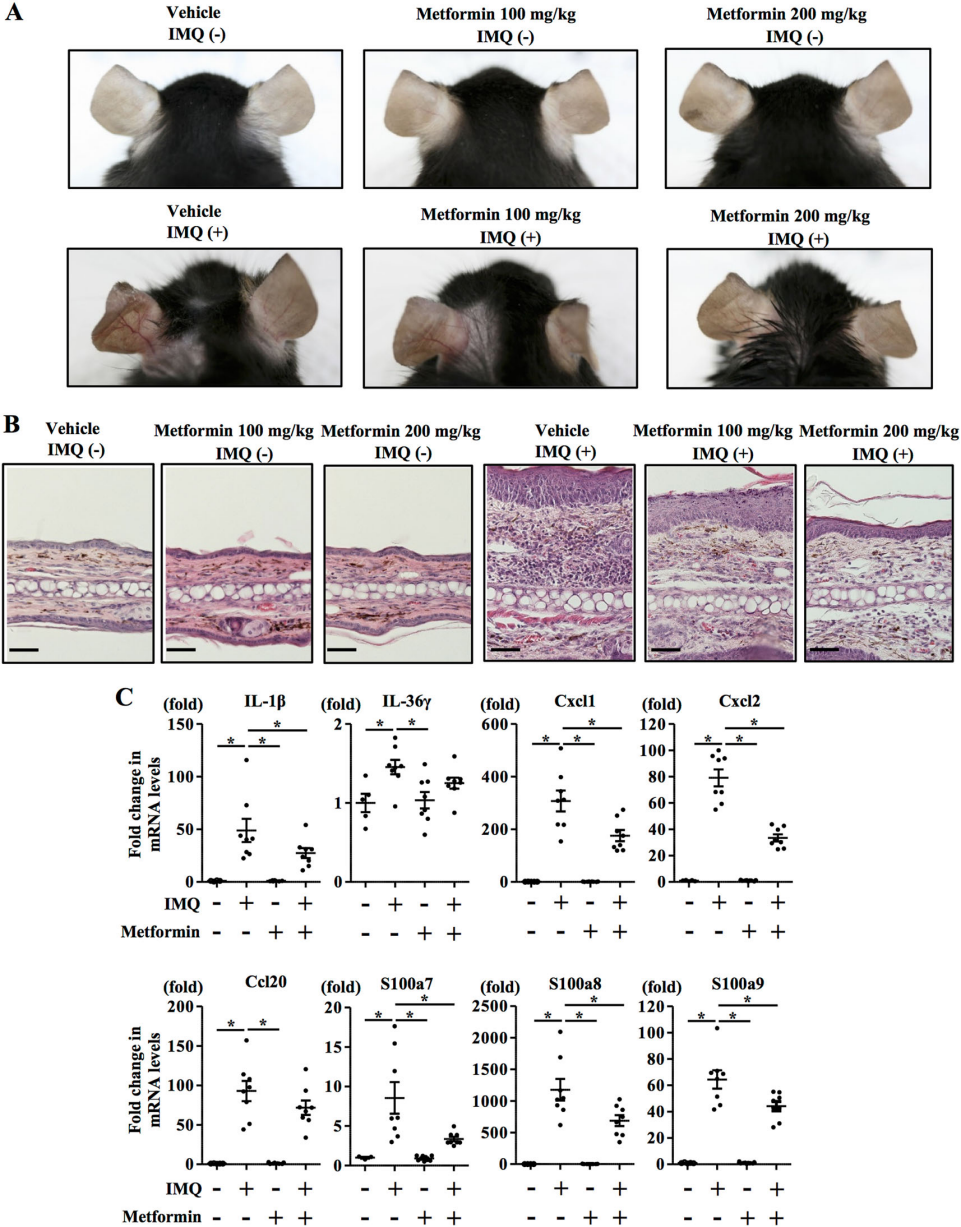
Oral metformin administration prevented the development of IMQ-induced psoriasiform eruptions. C57BL/6 mice were administered vehicle or metformin (100 and 200 mg/kg once daily) for 5 days from the day of topical application of IMQ to the left ear. **a** Clinical images of the ear. **b** Histology of the ear. Scale bar = 100 μm. **c** C57BL/6 mice were administered vehicle or metformin (200 mg/kg once daily) for 3 days from the day of topical application of IMQ to the ear. Then, mRNAs were extracted from the epidermis of the ear and IL-1β, IL-36γ, Cxcl1, Cxcl2, Ccl20, S100a7, S100a8 and S100a9 mRNA levels were analysed by qRT-PCR. **a**, **b** *n* = 6 for each group. **c** Data are expressed as mean ± SD; *n* = 6 for each group. *P < 0.05. **a**–**c** The data are representative of experiments repeated three times with similar results.

To further examine whether metformin treatment modifies the pathogenesis of psoriasis in vivo, we utilized a murine IMQ-induced psoriasiform eruption model. We administered either vehicle (distilled water once daily) or metformin (100 and 200 mg/kg once daily) to C57BL/6 mice for 5 days from the day of topical application of IMQ to the left ears. We evaluated the effect of the metformin treatment on the psoriatic lesions induced by IMQ through morphological observations and pathological examinations. Erythema, scaling and thickening were observed in the IMQ-induced skin lesions, in contrast to the findings in the vehicle-treated control group, whereas metformin treatment (200 mg/kg once daily) inhibited these pathological changes (Fig. 4a). Skin treated with IMQ demonstrated pathological changes of the epidermis, including acanthosis, parakeratosis and perivascular infiltration of inflammatory cells in the upper dermis. Metformin treatment (200 mg/kg once daily) reduced the thickness of the epidermis (Supplementary Fig. 3A and Fig. 4b) and the infiltration of inflammatory cells in IMQ-induced psoriatic lesions (Fig. 4b). Skin sections were also stained with anti-IL-17A antibody to assess the infiltration of IL-17A-producing cells, which is a hallmark of disease activity in psoriasis^31,46^. Metformin treatment (200 mg/kg orally, daily) significantly reduced IL-17A-producing cells in the dermis compared with the level in vehicle-treated control mice (Supplementary Fig. 3B); this infiltration was quantified by microscopic analysis of the skin sections.

Moreover, we analysed IL-1β, IL-36γ, Cxcl1, Cxcl2, Ccl20, S100a7, S100a8 and S100a9 mRNA expression in the epidermis of C57BL/6 mice administered either vehicle (distilled water once daily) or metformin (200 mg/kg once daily) orally for 3 days from the day of topical application of IMQ to the ears. The metformin treatment inhibited the upregulation of IL-1β, Cxcl1, Cxcl2, S100a7, S100a8 and S100a9 mRNA expression induced by the topical application of IMQ (Fig. 4c); in contrast, it did not significantly inhibit the upregulation of IL-36γ and Ccl20 mRNA expression. These findings suggest that metformin treatment prevented the development of psoriasiform eruptions in vivo.

**Fig. 5:**
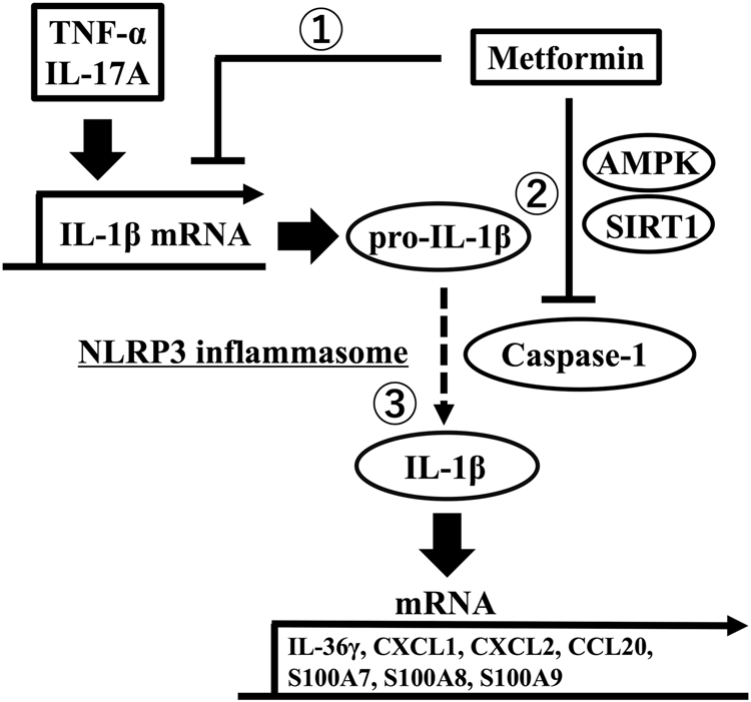
Metformin inhibits IL-1β secretion via impairment of NLRP3 inflammasome in keratinocytes. 1. Metformin inhibits upregulation of the IL-1β mRNA level induced by TNF-α and IL-17A stimulation, resulting in a reduced pro-IL-1β protein level. 2. Metformin downregulates caspase-1 protein level via AMPK and SIRT1. 3. Metformin inhibits TNF-α- and IL-17A-induced IL-1β secretion (mature IL-1β).

## Discussion

Psoriasis patients have been reported to frequently exhibit comorbidities such as T2DM^5–7^. Psoriasis patients with T2DM are frequently treated with metformin; however, whether such treatment directly affects the disease activity of psoriasis has remained untested. Indeed, clinical studies have shown that metformin treatment improves the severity of psoriasis and T2DM^22,23^.

As we did not utilize an IMQ-induced psoriasiform eruption model in mice with hyperglycaemia such as ob/ob mice, it could not be clarified whether metformin treatment contributes to prevent psoriasis development via the recovery of glucose metabolism. As skin inflammation in psoriasis alone can be a cause of hyperglycaemia in humans and IMQ-treated mice^47^, the effect of metformin on glucose metabolism is likely to be an important part of its anti-psoriatic effects. Considering that NLRP3 inflammasome activation is upregulated in patients with T2DM and metformin treatment decreases caspase-1 expression, and subsequently reduces IL-1β production in monocyte-derived macrophages from patients with T2DM^25^, our results should provide a comprehensive understanding of the role of metformin in the treatment of psoriasis patients with T2DM.

We have for the first time shown that metformin treatment lowered caspase-1 protein expression (Fig. 1). Caspase-1 is proteolytically activated from a proenzyme to produce a tetramer of its two active subunits, p20 and p10, which is necessary for efficient cytokine processing^48^. The total amount of caspase-1 protein expression is decreased in the course of cleavage of caspase-1. Therefore, there is a possibility that metformin treatment may increase the activated form of caspase-1 (p20 and p10 subunits); however, further studies will be needed to show that, as we did not examine the expression of p20 and p10 subunits. As it has been reported that cleavage of caspase-1 also has a mechanism to terminate inflammasome activity^48^, decreased caspase-1 protein expression induced by metformin treatment is likely to be involved in the inhibition of NLRP3 inflammasome activation in NHEKs.

Although the mechanism behind this remains incompletely understood, the induction of autophagy by AMPK and SIRT1 activation might be involved. Autophagy is a self-digestion process that facilitates the turnover of damaged proteins^49^. Several studies have revealed that metformin is a potent activator of the AMPK signalling pathway, which mediates the induction of autophagy^50–52^. Although it has been reported that metformin treatment induces the phosphorylation of AMPK in HaCaT cells^43^, an immortalized human keratinocyte cell line, whether it induces this in primary normal keratinocytes such as NHEKs, has not been tested. The present study has shown that metformin is an activator of AMPK in NHEKs.

Furthermore, it has been reported that metformin activates autophagy via SIRT1^53^, and that SIRT1 could modulate the inhibition of NLRP3 inflammasome activation^54,55^. Considering the evidence that AMPK, SIRT1 and autophagy are coordinately regulated^56^, and that autophagy induction negatively regulates NLRP3 inflammasome activation^57–59^, there is a possibility that autophagy through AMPK and SIRT1 activation may be involved in the lowered caspase-1 protein expression induced by metformin, contributing to the inhibition of NLRP3 activation and IL-1β secretion (mature IL-1β); however, to demonstrate this, further studies are required. Recently, the IL-1β-IL-1R signalling pathway controlled by NLPR3 inflammasome has been reported to play a critical role in the pathogenesis of psoriasis in humans and induced by IMQ in mice^60^. Our results show that metformin treatment inhibited IL-1β secretion in vitro and prevented the development of IMQ-induced psoriasis in vivo, suggesting that metformin administration may impair the IL-1β-IL-1R signalling pathway controlled by NLPR3 inflammasome, leading to the prevention of psoriasis.

Interaction between keratinocytes and immune cells such as T cells and dendritic cells (DCs) is crucial in the pathogenesis of psoriasis^4^. Therefore, it is also possible that metformin treatment may modulate the functions of T cells and DCs, contributing to preventing the development of psoriasiform eruptions in vivo. Indeed, metformin has been reported to inhibit the proliferation of antigen-activated T cells^61^. Furthermore, metformin was reported to reduce IL-17A production and the population of Th17 cells, which are critical factors in the development of psoriasis, in a murine experimental autoimmune encephalomyelitis model^62^. Moreover, metformin was shown to reduce the expression of major histocompatibility complex class I and II, and costimulatory factors in DCs^63^, leading to inhibition of the antigen-presenting ability of DCs. These findings suggest that metformin has immunomodulatory effects on T cells and DCs, as well as on keratinocytes, contributing to preventing the development of psoriasiform eruptions; however, further studies will be needed to reveal how metformin treatment modifies the functions of T cells and DCs in the IMQ-induced psoriasis model. In addition, metformin inhibits the activation of several signalling pathways such as mitogen-activated protein kinase^64^, mammalian target of rapamycin^43^ and nuclear factor-κB signalling^65^. This results in the inhibition of keratinocyte proliferation and pro-inflammatory cytokine production, which are characteristic features of keratinocytes in psoriasis. Our results suggest that metformin treatment downregulated the pro-IL-1β mRNA level, which is partially supported by a report describing that metformin inhibits cytokine/chemokine expression via interfering with the transcriptional activity of nuclear factor-κB signalling.

In conclusion, our study showed that metformin treatment inhibited TNF-α- and IL-17A-induced inflammatory responses of keratinocytes via blocking NLRP3 inflammasome activation in vitro (Fig. 5). Oral metformin treatment significantly attenuated IMQ-induced psoriasis-like inflammation in vivo. The therapeutic benefits could be partially attributed to its interference with IL-1β secretion (mature IL-1β) derived from keratinocytes. The modulation of keratinocytes by metformin treatment suggested that metformin could be a promising immunomodulatory agent in psoriasis patients with T2DM.

## Supplementary information

Supplementary Figure Legends

Supplemental Figure 1

Supplemental Figure 2

Supplemental Figure 3
