# Supplementary material for: Metformin inhibits IL-1β secretion via impairment of NLRP3 inflammasome in keratinocytes: implications for preventing the development of psoriasis
Source: Cell Death Discov. 2020 Mar 4;6:11. doi: 10.1038/s41420-020-0245-8 (PMC7055596; doi:10.1038/s41420-020-0245-8)
Supplement: Supplementary file 1 — Supplementary Figure Legends [file 41420_2020_245_MOESM1_ESM.docx]

**Supplementary Figure 1. Cell viability assay**

NHEKs were treated with metformin, dorsomorphin and sirtinol at the indicated doses for 24 h. WST-1 cell proliferation assay was performed to evaluate cell viability. Data are expressed as mean ± S.E.M.; n = 3 for each group. Statistically significant differences between control and treated NHEKs are presented: **P* < 0.05.

**Supplementary Figure 2. Primers for qRT-PCR**

**Supplementary Figure 3. Oral metformin administration prevented the development of IMQ-induced psoriasiform eruptions.**

C57BL/6 mice were administered vehicle or metformin (100 and 200 mg/kg, once daily) for 5 days from the day of topical application of IMQ to the ear. (A) Ear thickness was measured using a dial thickness gauge. (B) Skin sections from six mice per group were stained with an anti-IL-17A antibody and then secondary antibody, and photomicrographs were taken of representative 400× magnified fields. Quantification of cells stained with anti-IL-17A antibody in 400× magnified fields of the dermis was performed in a blinded manner (three fields were analysed per skin section). (A and B) Data are expressed as mean ± S.D.; n = 6 for each group. **P* < 0.05. The data are representative of experiments repeated three times with similar results.
