# Supplementary figures and images for: Metformin inhibits IL-1β secretion via impairment of NLRP3 inflammasome in keratinocytes: implications for preventing the development of psoriasis
Source: Cell Death Discov. 2020 Mar 4;6:11. doi: 10.1038/s41420-020-0245-8 (PMC7055596; doi:10.1038/s41420-020-0245-8)

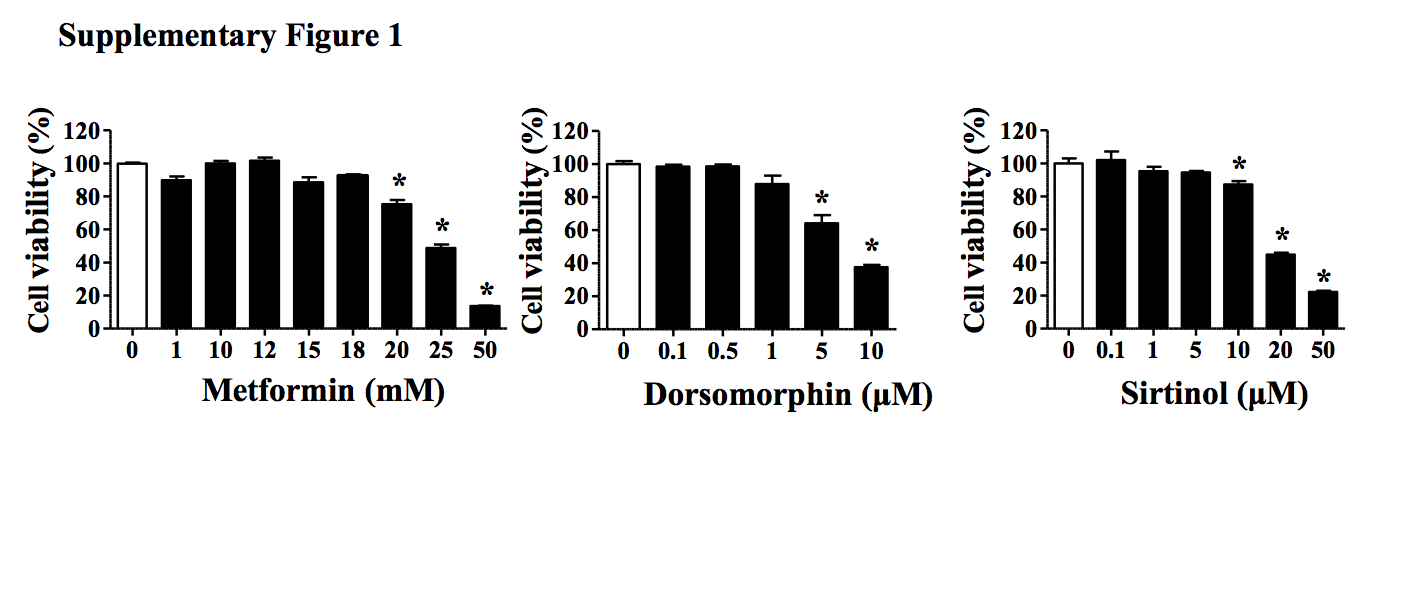

Supplement: Supplementary file 2 — Supplemental Figure 1 [file 41420_2020_245_MOESM2_ESM.tif]

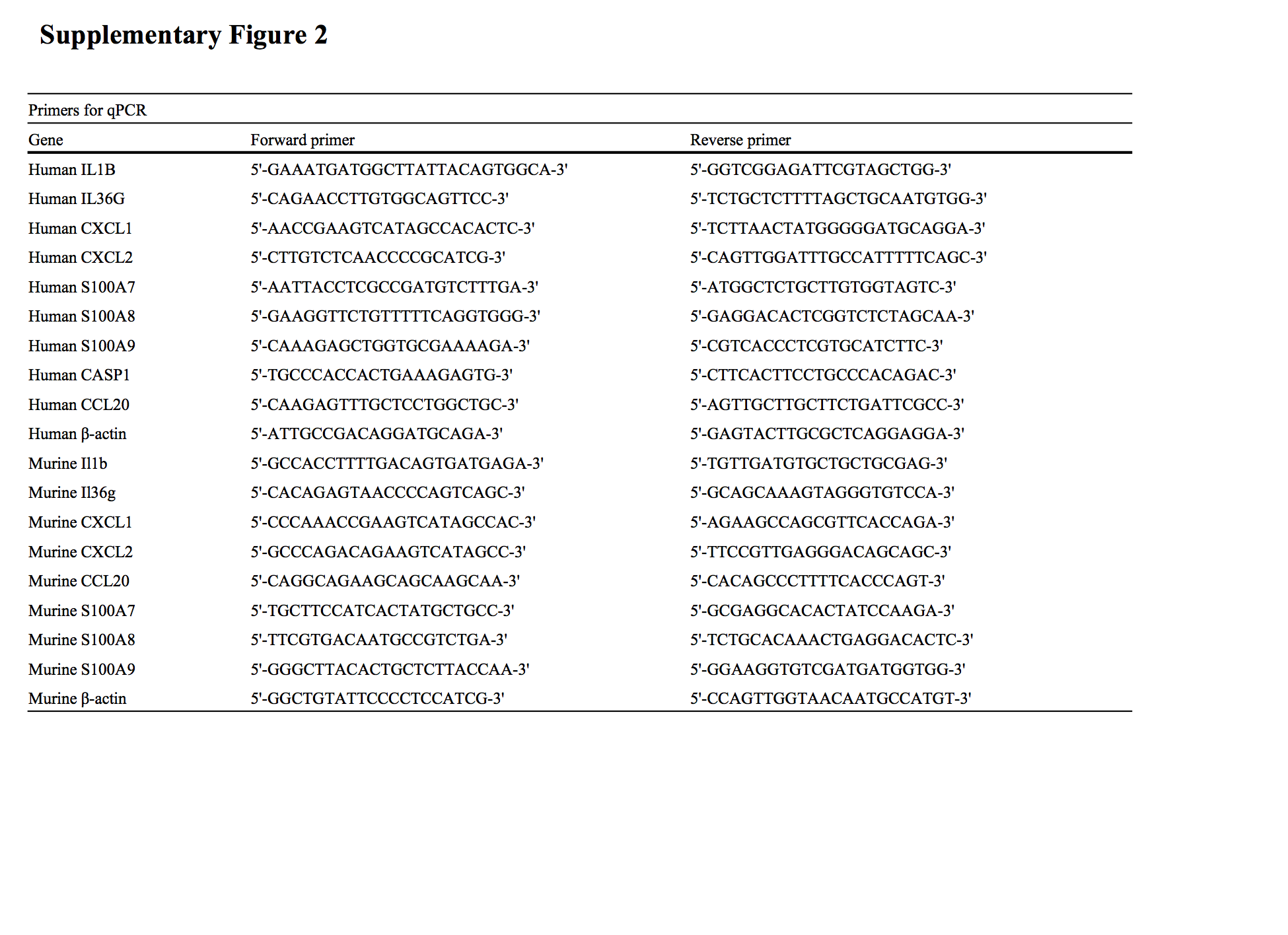

Supplement: Supplementary file 3 — Supplemental Figure 2 [file 41420_2020_245_MOESM3_ESM.tif]

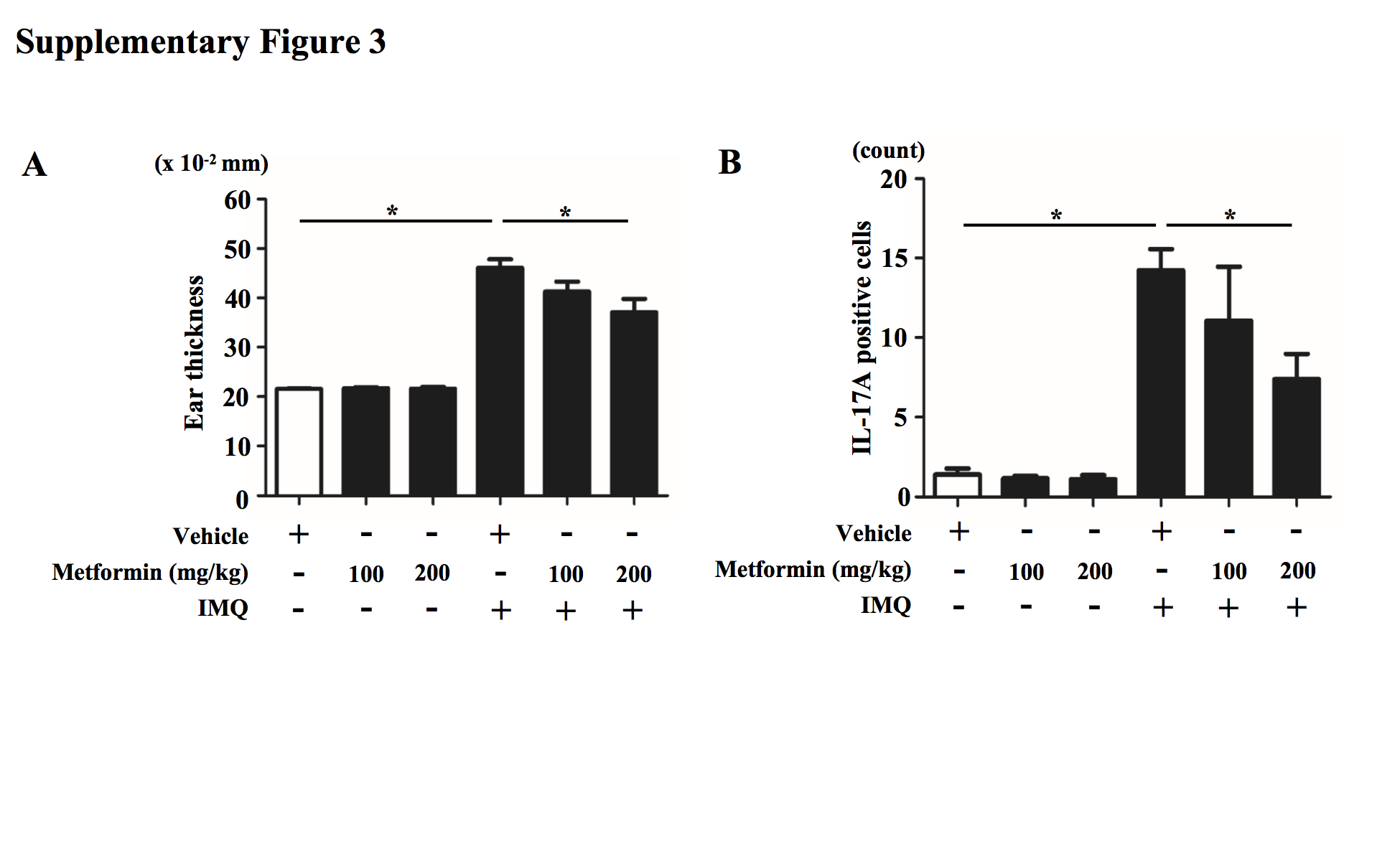

Supplement: Supplementary file 4 — Supplemental Figure 3 [file 41420_2020_245_MOESM4_ESM.tif]
